# Modeling and Control of a Wheeled Biped Robot

**DOI:** 10.3390/mi13050747

**Published:** 2022-05-08

**Authors:** Zemin Cui, Yaxian Xin, Shuyun Liu, Xuewen Rong, Yibin Li

**Affiliations:** 1 School of Control Science and Engineering, Shandong University, Jinan 250100, China; CZM199307@163.com (Z.C.); liyb@sdu.edu.cn (Y.L.); 2 School of Rail Transportation, Shandong Jiaotong University, Jinan 250357, China; 220103@sdjtu.edu.cn; 3 Department of Assets and Laboratory Management, Shandong University, Jinan 250100, China

**Keywords:** wheeled biped robot, linear quadratic regulator, model predictive control, model decoupling

## Abstract

It is difficult to realize the stable control of a wheeled biped robot (WBR), as it is an underactuated nonlinear system. To improve the balance and dynamic locomotion capabilities of a WBR, a decoupled control framework is proposed. First, the WBR is decoupled into a variable-length wheeled inverted pendulum and a five-link multi-rigid body system. Then, for the above two simplified models, a time-varying linear quadratic regulator and a model predictive controller are designed, respectively. In addition, in order to improve the accuracy of the feedback information of the robot, the Kalman filter is used to optimally estimate the system state. The control framework can enable the WBR to realize changing height, resisting external disturbances, velocity tracking and jumping. The results obtained by simulations and physical experiments verify the effectiveness of the framework.

## 1. Introduction

Wheels have the advantages of high efficiency and rapid movement, while leg-based locomotion is more versatile and can overcome challenging environments. Combining the advantages of the two can achieve the best of both worlds [1,2,3].

The hydraulically driven Handle robot [4] developed by Boston Dynamics can realize functions such as rapid acceleration and deceleration, turning, jumping, and handling heavy objects. The movement speed can reach 14.5 km/h, and the robot has excellent dynamic balance ability. Unfortunately, no specific details about Handle or its control system have been published. Since the introduction of Handle in 2017, many researchers around the world have been working on wheeled bipedal robots (WBRs). The hydraulic wheel-legged robot WLR [5] and the WLR-II [6] developed by HIT have effectively improved the reliability of the hydraulic system by building the oil circuit into the structure. The WLR can squat, move over rough terrain and carry heavy loads. Ascento [7], developed by ETH, successfully decouples jump and balance control using linkage mechanism, which improves system strength and reduces mass through topology optimization design. The weight of Ascento is only 10.4 kg, and it can achieve jump and fall recovery. Zhang et al. [8] designed a bipedal legged wheel robot SR600, and realized the balance control through a PID controller. Liu et al. [9] developed a wheel-biped transformable robot SR600-II and proposed a wheel and foot transformation strategy. Zhao et al. [10] designed a new electric wheel-legged humanoid robot BHR-W, which has capabilities of bipedal walking and wheel rolling. Unfortunately, the above mentioned studies mainly focus on structural design and experimental validation.

The WBR is an underactuated system and must be dynamically balanced by proper control methods. Raza et al. [11] proposed a scheme combining a nonlinear feedforward controller with a linear quadratic regulator (LQR) and verified it on the Hebi robot. Experiments showed that this method could achieve good trajectory tracking and interference suppression. Xin et al. [12] established a motion balance controller for a WBR based on the inverse dynamics and uncertainty and disturbance estimation (UDE) method, and verified the robustness of the method by simulations. Wang et al. [13] proposed an interconnection and damping assignment-passivity-based control (IDA-PBC) method, which can enable the WBR to obtain a wide range of stability. Nevertheless, the above methods all regard the WBR as a wheeled inverted pendulum, and do not consider the influence of the torso on the motion of the robot. Klemm et al. [14] proposed a method for LQR-assisted whole-body control, with which the Ascento robot can adapt to uneven terrain and be more robust to disturbances. Xin et al. [15] proposed a distributed whole-body dynamics modeling method and a whole-body control framework, under which the WBR can achieve rapid acceleration and deceleration while maintaining its own balance. Yet the modeling and computation of whole body control is too complex. Zhou et al. [16] proposed a decoupled control framework and designed a wheel balance controller and a center of mass (CoM) adjustment controller, respectively. The frame enables the WBR to achieve accurate trajectory tracking and to stand stably on slopes. Chen et al. [17] used the wheeled-spring-loaded inverted pendulum (W-SLIP) model to characterize the dynamics of the WBR and designed a hierarchical control scheme. The simulation results showed that the framework could make the WBR jump stably. However, the above two control schemes do not fully consider environmental constraints.

Predictive models have long been thought to play a role in the locomotion strategies of animals [18]. In recent years, owing to the development of computer hardware and optimization methods, model predictive control (MPC) has been widely used in quadruped robots [19] and biped robots [20]. The MPC-based controller can easily integrate various constraints and improve the stability of the robot by predicting the time of flight and underactuation [21,22]. Research has shown that a preview controller can be used to compensate the zero moment point (ZMP) error caused by the difference between a simple model and the precise multibody model [23]. In addition, ZMP preview control can improve the anti-interference ability of biped robots [24], which is also crucial for WBRs.

This paper proposes a new control framework for WBRs that has the advantages of simple modeling and strong robustness. Based on this framework, the WBR can realize various stable motions. In detail, the main contributions of this paper can be summarized as follows:

1. The WBR is decoupled into two simple models of a variable-length wheeled inverted pendulum (VL-WIP) and a five-link multi-rigid body system, which simplifies the modeling and control of the robot. The basic stability of the robot is achieved by using a time-varying linear quadratic regulator (TV-LQR). As a supplement, the translation of the upper body of the robot is realized through MPC. In the process of constructing the MPC controller, the dynamic stability principle of the robot is fully considered.

2. The optimal state estimation of the WBR is realized by linear Kalman filtering, which reduces the influence of sensor noise on the robot and improves the robustness of the controller.

3. The results of experiments conducted on a physical prototype verify the effectiveness of the control framework proposed in this paper.

The rest of this article is organized as follows. Section 2 gives the overview of the WBR. The dynamic modelling is introduced in Section 3. Section 4 presents the control strategy. Experimental results obtained from simulations and physical tests are described in Section 5. Section 6 contains conclusions and future work.

## 2. Overview of WBR

**Figure 1:**
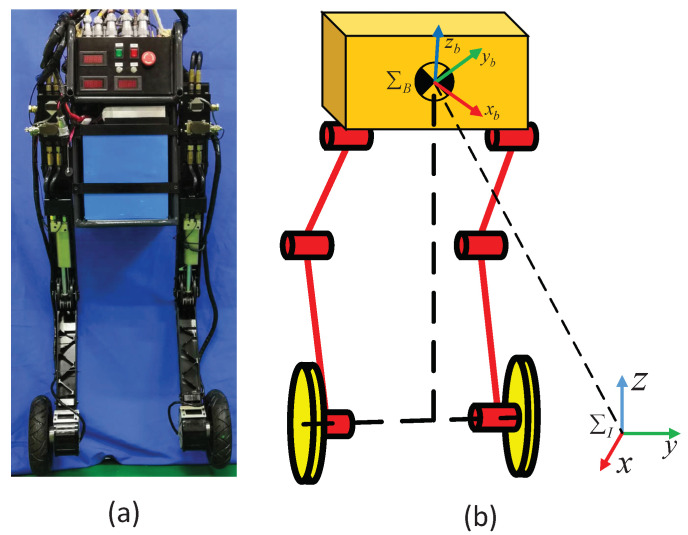
Model of the wheeled biped robot (WBR): (**a**) Scooter robot prototype; (**b**) simplified model of Scooter.

The research focus of this work was to solve the balance and motion control problems of wheeled biped robots. As shown in Figure 1a, the research object of this work was a WBR named Scooter, which was constructed by Shandong University, and its simplified model is shown in Figure 1b. In the Figure 1b, $\Sigma_{I}$ represents the inertial system fixed to the ground, and $\Sigma_{B}$ represents the 6-DoF floating base coordinate system of the robot. Scooter is a hydraulic WBR that consists of a floating base body and two legs with driving wheels at ends. Each leg has 3 degrees of freedom, in which the hip and knee joints are driven by hydraulic actuators and the wheels are driven by DC brushless motors. Hydraulic actuators are controlled by servo valves and integrate displacement and force sensors for feedback of position and force information. An encoder is installed on the motor for feedback of the angle and speed of the wheel. The wheels are driven directly by an electric motor without a gearbox, which helps absorb shocks from the ground. The wheel motor feedback torque information through proprioception, without using any force sensor and torque sensor [25]. The knee joint uses a four-bar linkage to increase the range of motion. A high-performance embedded controller and an inertial measurement unit (IMU) for detecting the robot pose are mounted on the torso. The total mass of the Scooter is 80 kg, including the battery and the onboard hydraulic power unit. During the calculations in later chapters, all kinetic parameters are exactly the same as the physical prototype.

## 3. Dynamic Modeling

**Figure 2:**
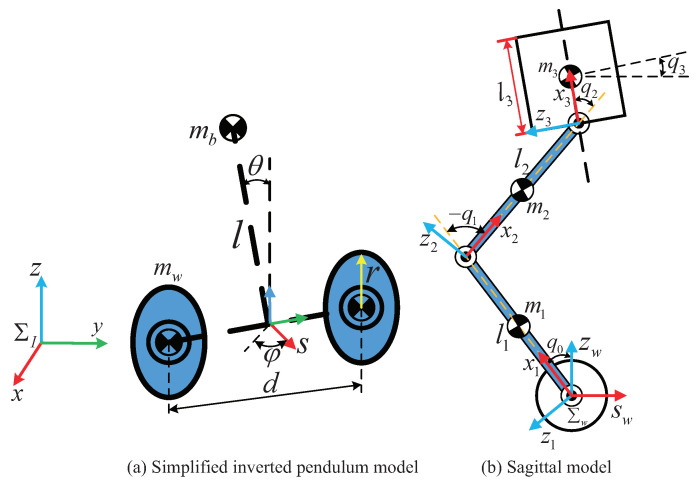
Decoupled model: the VL-WIP model and the floating base upper body mode (sagittal model). $\Sigma_{w}$ represents the axle coordinate system.

To reduce the difficulty of modeling and control, the WBR is decoupled into two simplified models, and then the required torques of the joints are determined by their respective controllers. The decoupling model is depicted in Figure 2. The WBR is decoupled as a VL-WIP model and a floating base five-link multi-rigid body system. Next, we will model the two parts separately. Since the left and right sides of the robot are completely symmetrical, and the legs only have pitch degrees of freedom. For brevity, we only show the sagittal plane model here. The parameters of the decoupling model are shown in Table 1.

### 3.1. Equivalent Centroid Calculation

In the decoupling process, the five-link multi-rigid body system is equivalent to a lumped mass point. The position of the equivalent centroid is weighted by the masses of the individual links and their centroid positions. To establish the relationship between this center of mass and the axle coordinate system, the Denavit–Hartenberg (D-H) convention was used to establish the kinematic model. The homogeneous transformation matrix between the coordinate system *i* and the axle coordinate system is as follows:

(1) Tiw=RiwPiw001

where Riw is the orientation matrix of the coordinate system *i* relative to the axle coordinate system, Piw is the position matrix of the coordinate system *i* relative to the axle coordinate system.

The position of the CoM of the upper body relative to the axle coordinate system can be obtained as

(2) PCw(q)=∑i=1nmi·PCiw(q)∑i=1nmiPCiw(q)=Tiw(q)·PCii

where $m_{i}$ is the mass of the *i*-th link, $q={\left[q_{1}\;q_{2}\;q_{3}\right]}^{T}$ is the actual angle of the sensor feedback, PCw=[SCZC]T is the position coordinate of the equivalent CoM relative to the axle coordinate system, PCiw is the position coordinate of the CoM of the *i*-th link in the wheel axis coordinate system, and PCii is the position of the CoM of the *i*-th link in the local coordinate system.

According to the coordinates of CoM, the pendulum length *l* and inclination angle θ of the inverted pendulum can be obtained.

(3) $$\begin{array}{c} l=\sqrt{S_{C}^{2}+Z_{C}^{2}}\\ \theta =atan(\frac{S_{C}}{Z_{C}}) \end{array}$$

### 3.2. VL-WIP Modeling

The classic two-wheeled inverted pendulum model has recently been studied in the literature [26]. Different from literature [26], the pendulum length of the inverted pendulum involved in this paper is variable. $\phi ={\left[s\;\theta \;\varphi \right]}^{T}$ is selected as the generalized coordinates of the VL-WIP. The dynamics equations of the VL-WIP can be obtained via Euler–Lagrange equations, as given below:

(4) $$M\left(\phi \right)\ddot{\phi }+C\left(\phi ,\dot{\phi }\right)=B\tau_{w}$$

where *M*, *C* and *B* represent the generalized inertial matrix, the Coriolis force, Centripetal force, and gravitational force matrix, and the input matrix, respectively. $\tau_{w}={\left[\tau_{l}\;\tau_{r}\right]}^{T}$ is the wheel driving torque. After calculation,

(5) $$\begin{array}{cc} \; & M\left(\phi \right)=\left[\begin{array}{ccc} m_{b}+2m_{w}+2\frac{I_{w}}{r^{2}} & m_{b}lcos\theta & 0\\ m_{b}l\cos\theta & m_{b}l^{2}+I_{y} & 0\\ 0 & 0 & \frac{d^{2}m_{w}}{2}+\frac{d^{2}I_{w}}{2r^{2}}+I_{z} \end{array}\right]\\ \; & C\left(\phi ,\dot{\phi }\right)=\left[\begin{array}{c} -m_{b}l{\dot{\theta }}^{2}\sin\theta \\ -m_{b}gl\sin\theta \\ 0 \end{array}\right]\\ \; & B=\left[\begin{array}{cc} r^{-1} & r^{-1}\\ -1 & -1\\ \frac{d}{2r} & -\frac{d}{2r} \end{array}\right] \end{array}$$

### 3.3. Modeling of the Multi-Rigid Body System

To improve computational efficiency, a simplified single rigid-body model with lumped mass and inertia is considered to characterize main dynamics of the upper body. In this paper, we limit the application of the upper body to sagittal motion. The yaw control of the robot is realized by the differential motion of the two wheels, and the pitch angle of the torso is controlled by the hip joint. The roll angle of the WBR can be controlled by adjusting the height of the two legs, but in this paper, both legs perform the same motion, so the roll angle is always kept at zero. To sum up, we only need to model the translational dynamics of the upper body.

**Figure 3:**
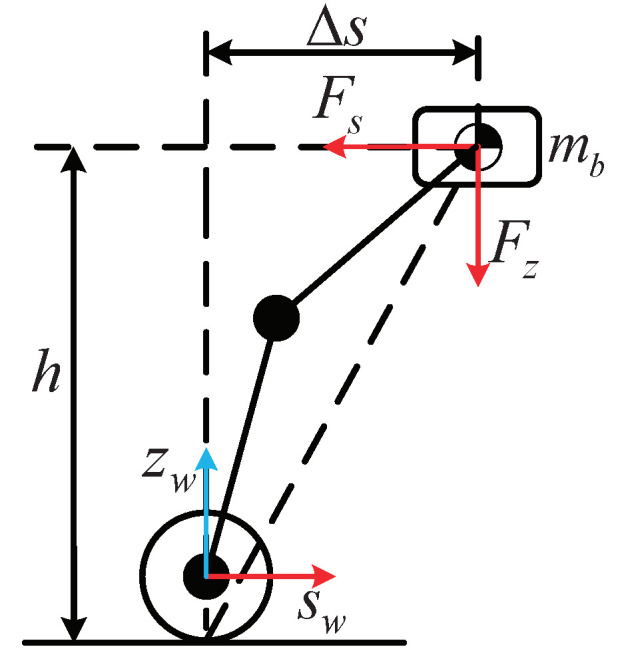
Stability analysis of the lumped mass model. Δs is the horizontal offset of the CoM relative to the wheel. *h* is the vertical height of the CoM, which can be calculated from the leg kinematics. $F_{s}$ and $F_{z}$ represent horizontal inertial force and vertical inertia force, respectively.

As introduced in literature [27], in order to ensure the dynamic stability of the inverted pendulum, the resultant force acting on the CoM, which consists of gravity and inertial force, must point to and pass through the supporting polygon. The schematic diagram is depicted in Figure 3.

From the previous analysis, we know that to ensure that the robot does not overturn, the inertial force applied at the CoM should satisfy the following relationship.

(6) $$\frac{F_{s}}{\Delta s}=\frac{F_{z}}{h}$$

where $F_{s}=m\ddot{s}$, $F_{z}=m\left(g+\ddot{z}\right)$, in which, $\ddot{s}$ is the horizontal acceleration of the CoM, and $\ddot{z}$ is the vertical acceleration of the CoM.

According to Equation (6), we can derive the following equation as

(7) $$\ddot{s}=\frac{g+\ddot{z}}{h}\Delta s$$

In the vertical direction, the WBR must maintain the required standing height. The rigid body dynamics in the vertical direction is given by

(8) $$\ddot{z}=\frac{F_{z}}{m_{b}}-g$$

The simplified robot dynamics can be combined into the following form:

(9) $$\left[\begin{array}{c} \dot{s}\\ \ddot{s}\\ \dot{z}\\ \ddot{z}\\ 0 \end{array}\right]=\left[\begin{array}{ccccc} 0 & 1 & 0 & 0 & 0\\ 0 & 0 & 0 & 0 & 0\\ 0 & 0 & 0 & 1 & 0\\ 0 & 0 & 0 & 0 & 1\\ 0 & 0 & 0 & 0 & 0 \end{array}\right]\left[\begin{array}{c} s\\ \dot{s}\\ z\\ \dot{z}\\ -g \end{array}\right]+\left[\begin{array}{cc} 0 & 0\\ \frac{g+\ddot{z}}{h} & 0\\ 0 & 0\\ 0 & \frac{1}{m_{b}}\\ 0 & 0 \end{array}\right]\left[\begin{array}{c} \Delta s\\ F_{z} \end{array}\right]$$

To facilitate subsequent derivations, Equation (9) can be expressed in standard state space form:

(10) $$\dot{x}\left(t\right)=A_{c}x\left(t\right)+B_{c}u\left(t\right)$$

## 4. Control Strategy

**Figure 4:**
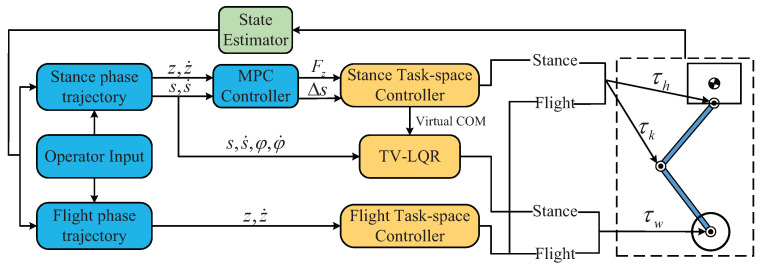
Overview of the control architecture. Blocks shaded blue run at 100 Hz, blocks shaded green run at 500 Hz, and blocks shaded gold run at 1000 Hz.

The basic control framework of the system is depicted in the block diagram in Figure 4. A composite controller was created to determine the torques of the wheels and upper body joints respectively. The operator provides high-level commands by giving a desired translational velocity and turning rate. The displacement command is obtained by integrating. Various controllers use operator-entered commands and estimated robot states to generate torque commands for wheels and leg joints control. The following sections describe the implementation of the major components of the system on the WBR.

### 4.1. VL-WIP Controller

Because it effectively solves the problem of achieving a balance between good system response and control effort, LQR controller is widely used in WIP control. For the WBR, the pendulum length is constantly changing during movement, so the TV-LQR controller is used to control the wheels. The state vector was defined as $X={\left[s\;\theta \;\varphi \;\dot{s}\;\dot{\theta }\;\dot{\varphi }\right]}^{T}$. By solving the kinetic Equation (4), we can obtain the following equation:

(11) $$\begin{array}{cc} \; & \dot{X}=f\left(X,l,U\right) \end{array}$$

After linearization, the dynamics model of VL-WIP can be given by

(12) $$\dot{X}=A\left(l\right)X+B\left(l\right)U$$

where A(l) and B(l) are the linearized matrices.

(13) $$\begin{array}{ccc} \; & A\left(l\right)=\left[\begin{array}{cccccc} 0 & 0 & 0 & 1 & 0 & 0\\ 0 & 0 & 0 & 0 & 1 & 0\\ 0 & 0 & 0 & 0 & 0 & 1\\ 0 & a_{1} & 0 & 0 & 0 & 0\\ 0 & a_{2} & 0 & 0 & 0 & 0\\ 0 & 0 & 0 & 0 & 0 & 0 \end{array}\right]\; & B\left(l\right)=\left[\begin{array}{cc} 0 & 0\\ 0 & 0\\ 0 & 0\\ b_{1} & b_{1}\\ b_{2} & b_{2}\\ b_{3} & -b_{3} \end{array}\right]\\ \; & U=\left[\begin{array}{c} \tau_{l}\\ \tau_{r} \end{array}\right] \end{array}$$

where

(14) $$\begin{array}{cc} \; & a_{1}=-\frac{gl^{2}m_{b}^{2}r^{2}}{2I_{w}\left(I_{y}+m_{b}l^{2}\right)+\left(2l^{2}m_{b}m_{w}+I_{y}\left(m_{b}+2m_{w}\right)r^{2}\right)}\\ \; & a_{2}=\frac{glm_{b}\left(2I_{w}+\left(m_{b}+2m_{w}\right)r^{2}\right)}{2I_{w}\left(I_{y}+m_{b}l^{2}\right)+\left(2l^{2}m_{b}m_{w}+I_{y}\left(m_{b}+2m_{w}\right)r^{2}\right)}\\ \; & b_{1}=\frac{r(I_{y}+lm_{b}\left(l+r\right))}{2I_{w}\left(I_{y}+m_{b}l^{2}\right)+\left(2l^{2}m_{b}m_{w}+I_{y}\left(m_{b}+2m_{w}\right)r^{2}\right)}\\ \; & b_{2}=-\frac{2I_{w}+r\left(lm_{b}+\left(m_{b}+2m_{w}\right)r\right)}{2I_{w}\left(I_{y}+m_{b}l^{2}\right)+\left(2l^{2}m_{b}m_{w}+I_{y}\left(m_{b}+2m_{w}\right)r^{2}\right)}\\ \; & b_{3}=\frac{dr}{2I_{z}r^{2}+d^{2}\left(I_{w}+m_{w}r^{2}\right)} \end{array}$$

From the state space equation, it is known that the forward control and steering control of WIP are completely decoupled. The goal of the TV-LQR method is to obtain a state feedback matrix K, which minimizes the infinite-time quadratic performance index:

(15) $$J\left(\tilde{X},U\right)=\int_{0}^{\infty }\left[{\tilde{X}}^{T}Q\tilde{X}+U^{T}RU\right]dt$$

where $\tilde{X}=X-X^{ref}$ is the error vector of system state, $X^{ref}$ is the target state, *Q* and *R* are weight matrices.

The torque profile at the wheel actuators is determined by

(16) $$U=-K\tilde{X}$$

### 4.2. Upper-Body Controller

In order to improve the robustness of control, MPC is used to solve the horizontal displacement of the CoM and the vertical force. It can predict the state variation of the WBR in a longer time frame. For the convenience of computer solution, Equation (10) is expressed in discrete-time form

(17) $$x_{k+1}=A_{k}x\left(k\right)+B_{k}u\left(k\right)$$

where $A_{k}=I+A_{c}\cdot \Delta T$, $B_{k}=B_{c}\cdot \Delta T$, in which ΔT is the discretized time interval.

It can be formulated to plan input *u* to minimize the cost function

(18) $$J\left(x,u\right)=\sum_{i=0}^{k}\left[{\left(x_{i}-x_{i}^{d}\right)}^{T}S_{i}\left(x_{i}-x_{i}^{d}\right)+u_{i}^{T}W_{i}u_{i}\right]$$

such that:

$$\begin{array}{c} -\mu h\leq \Delta s\leq \mu h,\\ -\sqrt{L_{max}^{2}-z_{b}^{2}}\leq \Delta s\leq \sqrt{L_{max}^{2}-z_{b}^{2}},\\ F_{min}\leq F_{z}\leq F_{max} \end{array}$$

where $x_{i}$ is the robot’s state at time step *i*, $x_{i}^{d}$ is the desired state of the robot, $S_{i}$ and $W_{i}$ are the state following weight and input weight, respectively, $u_{i}$ is the input at time step *i*, and *k* is the horizon length. μ is the friction coefficient between the wheel and the ground. $L_{max}$ is the limit length of the leg. $z_{b}$ represents the vertical distance between the axle and the hip joint. These constraints limit the square pyramid approximation of the friction cone, leg work space, and the minimum and maximum z-force.

To ensure the solution efficiency, referring to the literature [28], the above optimal control problem is transformed into a quadratic programming. Once the optimal centroid horizontal displacement and vertical force are obtained, the next step is to map them into joint space. In our controller, virtual model control [29] is used to implement this function.

During the flight phase, the wheels of the robot remain motionless. To achieve the desired ground clearance, the position of the wheels relative to the base coordinate system needs to be planned. At this stage, virtual model control is still used for trajectory tracking. In addition, the torque calculated by the inverse dynamics is used as a feedforward term, thereby improving the tracking accuracy. The control law used to compute joint torques for the leg is

(19) $$\tau =J^{T}\left[k_{p}\left(p_{d}-p_{f}\right)+k_{d}\left(v_{d}-v_{f}\right)\right]+\tau_{ff}$$

where *J* is the leg Jacobian matrix, $k_{p}$ and $k_{d}$ are the virtual stiffness and damping matrices respectively. $p_{d}$ and $v_{d}$ are the desired position and velocity of the wheel in the base coordinate system, respectively. $p_{f}$ and $v_{f}$ are the actual position and velocity of the wheel in the base coordinate system, respectively. $\tau_{ff}$ is the feedforward torque.

### 4.3. State Estimation

The wheel odometer is easily affected by uneven terrain and wheel slippage, which leads to deviations in counting. Furthermore, the wheel encoder does not work during the jump of the robot. And the IMU calculates the position and speed of the wheel through integration. As time increases, a relatively large cumulative error will appear. Bloesch et al. [30] proposed to estimate the state of quadruped robots through extended Kalman filtering. This method has also been successfully applied to the wheeled biped robot Ascento [14]. However, this method is computationally expensive. In this article, the linear Kalman filter [31] is used to estimate the position and velocity of the WBR. The state equation of the Kalman filter is

(20) $${\left[\begin{array}{c} P_{b}\\ V_{b} \end{array}\right]}_{k+1}=\left[\begin{array}{cc} I_{2\times 2} & \Delta tI_{2\times 2}\\ 0_{2\times 2} & I_{2\times 2} \end{array}\right]{\left[\begin{array}{c} P_{b}\\ V_{b} \end{array}\right]}_{k}+\left[\begin{array}{c} 0_{2\times 2}\\ \Delta tI_{2\times 2} \end{array}\right]{\left[\begin{array}{c} a_{b} \end{array}\right]}_{k}+\omega_{k}$$

where $P_{b}\in R^{2\times 1}$ and $V_{b}\in R^{2\times 1}$ represent the position and velocity of the torso in the inertial frame, $a_{b}\in R^{2\times 1}$ is the acceleration at the torso measured by the IMU, Δt is the control period, and $\omega_{k}\in R^{4\times 1}$ is the system process noise.

The observation equation of the Kalman filter is

(21) $${\left[\begin{array}{c} P_{w}{+}^{w}P_{b}\\ V_{w}{+}^{w}V_{b} \end{array}\right]}_{k}=\left[\begin{array}{cc} I_{2\times 2} & 0_{2\times 2}\\ 0_{2\times 2} & I_{2\times 2} \end{array}\right]{\left[\begin{array}{c} P_{b}\\ V_{b} \end{array}\right]}_{k}+v_{k}$$

where $P_{w}\in R^{2\times 1}$ and $V_{w}\in R^{2\times 1}$ represent the position and velocity of the axle in the inertial frame, ${}^{w}P_{b}$ and ${}^{w}V_{b}$ are the position and velocity of the torso relative to the axle coordinate system, respectively. $v_{k}\in R^{4\times 1}$ is the observation noise.

Once the state and observation equations of the system are obtained, the state of the system can be estimated using the standard Kalman filter recursive equations. Notably, during the jumping process, the torso position error calculated based on the leg encoder is relatively large. At this time, to reduce the contribution of the legs to the odometer information, the observation noise is set to a larger value.

## 5. Simulation and Experiment

This section describes the validation of the effectiveness of the proposed control framework through four simulations, including changing height, resisting external disturbances, velocity tracking and jumping. Results from the physical prototype experiments of changing height and velocity tracking are also reported. Videos in Supplementary Materials of these simulations and experiments are available as attachments to this article.

### 5.1. Simulations

The overall control framework is validated using the open-source Webots simulation software. The simulation model has the same size and inertial parameters as the physical platform shown in Figure 1a. The torque limits and range of motion of all joints are also exactly the same as the actual platform. The robot is initialized at a vertically balanced configuration with standing height (vertical distance between hip joint and axle) at 0.6 m. For WBRs, the balance control of the sagittal plane is the core of the entire motion controller. Therefore, the motion of the robot in the sagittal plane was mainly studied in this paper.

#### 5.1.1. Changing Height

**Figure 5:**
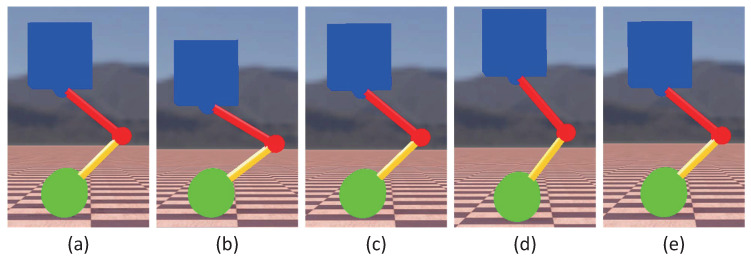
Snapshots of the simulation for the changing height. The standing height of the robot followed a sinusoidal trajectory. The photos (**a**,**c**,**e**) correspond to the normal standing height of the robot. Photos (**b**,**d**) correspond to the bottom and apex of the robot motion process, respectively.

**Figure 6:**
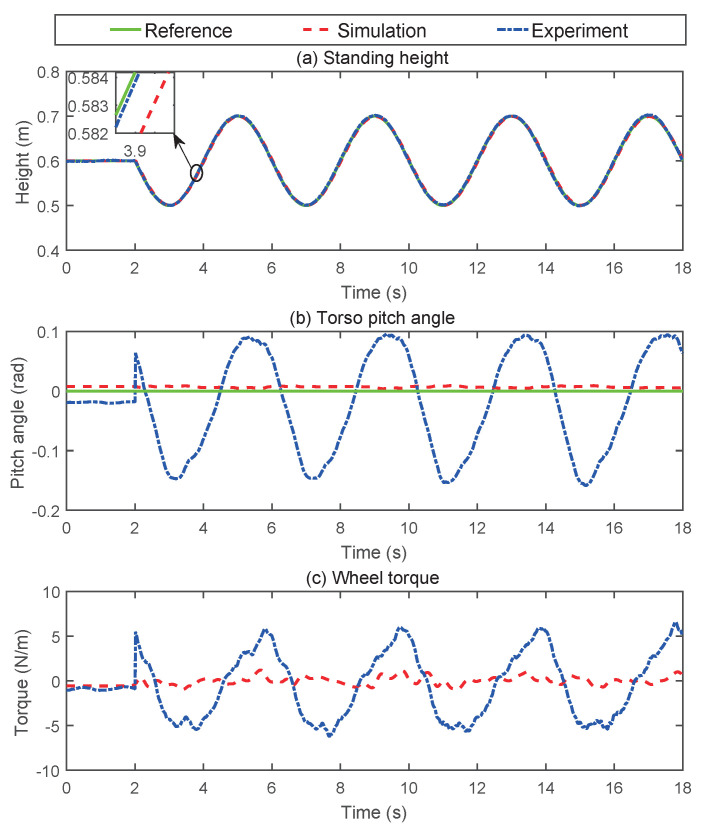
Changing height simulation data curve. (**a**) Stance height tracking trajectory. (**b**) The measurement of the torso pitch angle. (**c**) The actual driving torque of the wheel.

To verify the effectiveness of the control strategy proposed in the previous section, a variable-height simulation was first performed. Figure 5 presents snapshots of the simulation for the changing height. Figure 6 gives the data curve of the changing height simulation. As indicated in Figure 6, the robot can maintain balance while its standing height was constantly changing. The pitch angle of the torso varies in the range of 0.005 rad. In the process of changing the height, the wheel torque fluctuated because the parameter *l* of the TV-LQR controller changed.

#### 5.1.2. Sagittal Impact Recovery

**Figure 7:**
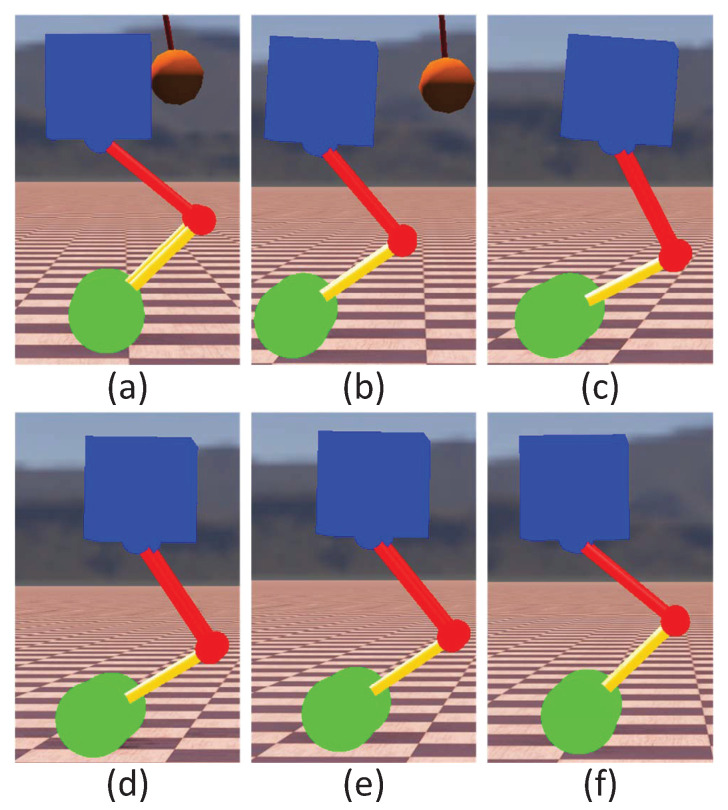
Snapshots of the simulation for the impact recovery. Photos (**a**–**c**) show that the robot maintains its balance by changing its state during the initial stage of being impacted. Photos (**d**–**f**) show the process of the robot gradually returning to the reference state.

**Figure 8:**
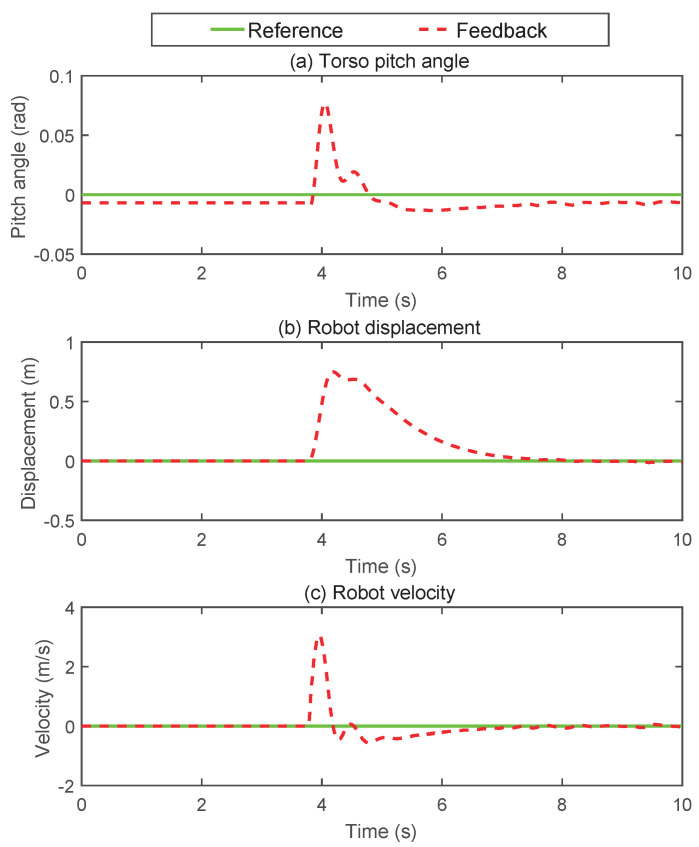
Sagittal plane impact adjustment process curve. (**a**) The measurement of torso pitch angle. (**b**) The displacement curve of the robot. (**c**) The velocity profile of the robot.

Shock resistance is an important indicator to characterize the stability of the WBR. To test the controller’s ability to suppress external disturbances, a spherical pendulum weighing 50 kg hit the robot’s torso at a speed of 1.8 m/s. The adjustment process after impact is shown in Figure 7. The state change of the robot is shown in Figure 8. It is noted from Figure 8 that the disturbance caused the speed and displacement of the robot to deviate from the expected values, and the pitch angle of the torso also fluctuated briefly. Under the regulation of the composite controller, the robot regains stability after being perturbed by about 4 s. It could be seen that the proposed control method had good robustness to external disturbances.

#### 5.1.3. Velocity Tracking

**Figure 9:**
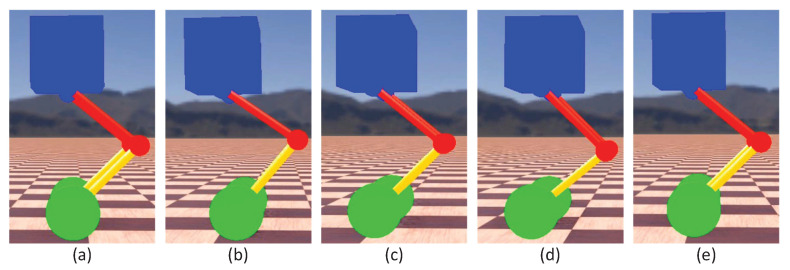
Snapshots of the simulation for the velocity tracking. Photos (**a**,**e**) represent the initial standing state and final stopped state of the robot, respectively. Photo (**b**) shows the process of accelerated motion of the robot. Photo (**c**) shows the state of the robot moving at a constant speed. Photos (**d**) represent the deceleration phase of the robot.

**Figure 10:**
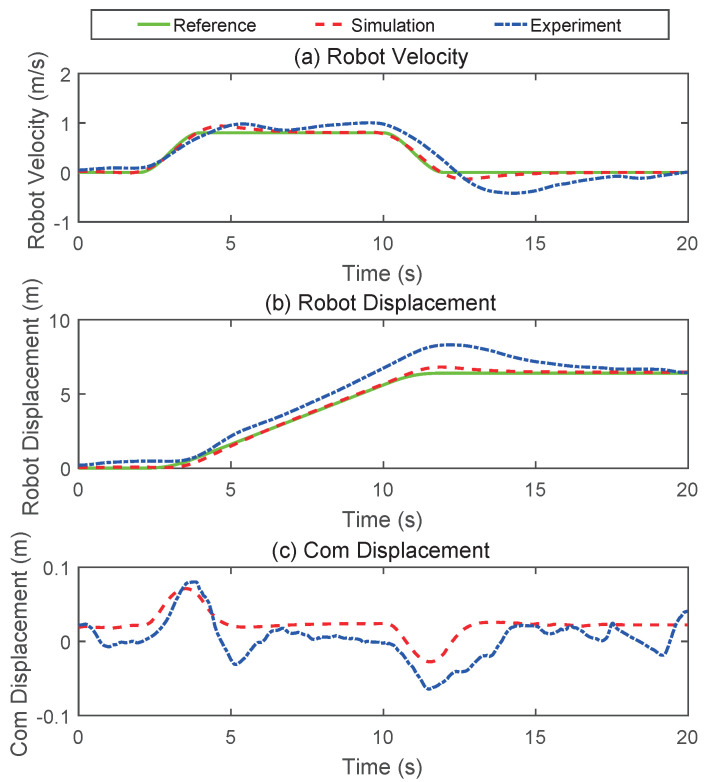
The simulation results of velocity tracking. (**a**) The velocity profile of the robot. (**b**) The displacement profile of the robot. (**c**) The horizontal displacement of the centroid.

The control objective of the velocity tracking simulation is to follow the desired reference velocity profile while maintaining the desired attitude and standing height. The whole motion process is divided into three stages: acceleration, constant speed and deceleration. The maximum speed and acceleration of the robot during the simulation were 0.8 m/s and 0.6 m/s${}^{2}$, respectively. As shown in Figure 9 and Figure 10c, in the process of the WBR acceleration, the CoM of the robot translated forward, and during the deceleration process, the CoM of the robot translated backward, and the position of the CoM remained unchanged during the uniform motion stage. The simulation phenomenon is consistent with the conclusion in Section 3.3. This showed that under the action of the composite controller, the robot would adaptively adjust its posture to maintain its balance. It was noted from Figure 10a that at the end of acceleration and deceleration, the speed had a slight overshoot. This was attributed to the excessive inertia of the torso.

#### 5.1.4. Jumping

**Figure 11:**
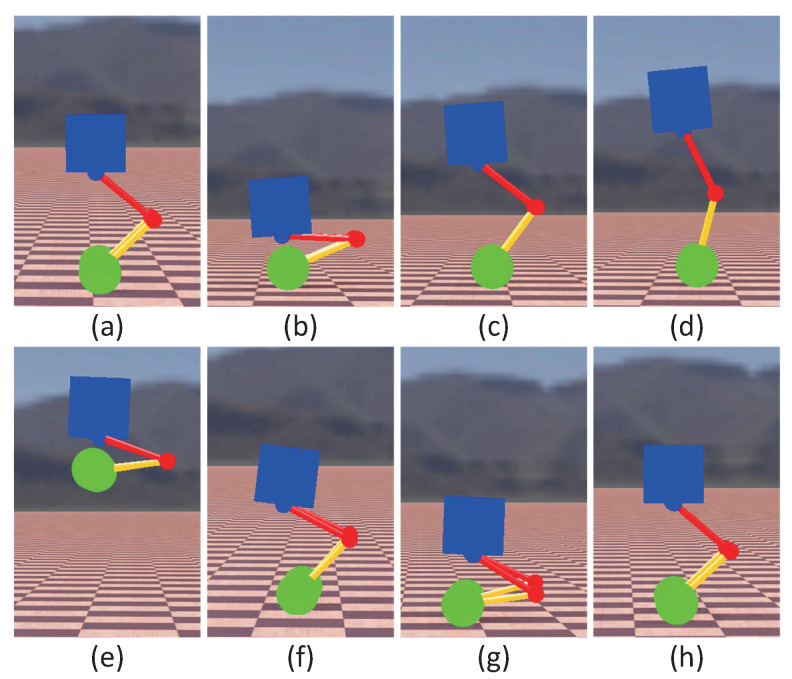
Snapshots of the simulation for the jumping. Photos (**a**,**b**) represent the squatting process of the robot. Photos (**c**,**d**) represent the take-off process of the robot. Photo (**e**) shows the state of the robot at the vertex. Photos (**f**,**g**,**h**) show the process of the robot landing.

**Figure 12:**
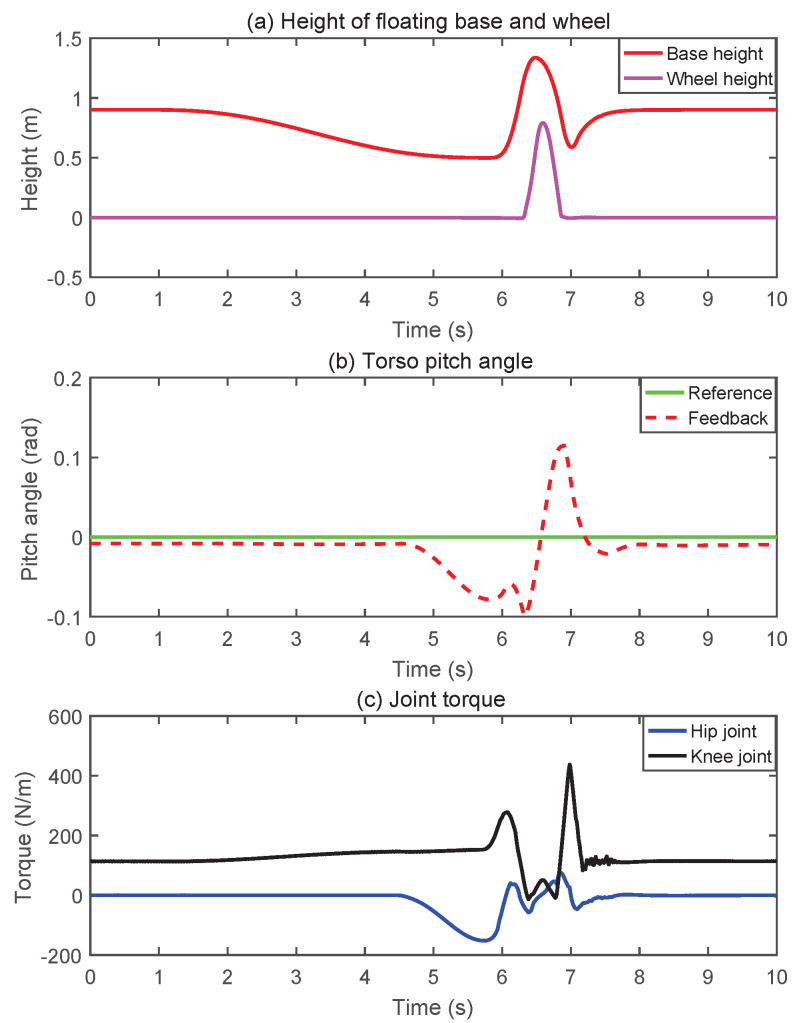
The simulation results of jumping. (**a**) Trajectories of the floating base and wheels. (**b**) The measurement of the torso pitch angle. (**c**) The profile of the joint torque.

Jumping mainly includes four stages: squatting, takeoff, swinging legs in the air and landing. Figure 11 presents snapshots of the simulation for the jumping. Figure 12 shows simulation results of jumping. As shown in Figure 12a, the height of the wheels from the ground could reach 0.8 m during the robot jumping. The drop in torso height after landing is because the robot absorbs the impact from the ground through the leg cushions, which is done autonomously by the MPC controller. As shown in Figure 12b, when landing, owing to the impact of the ground, the robot’s torso would swing briefly, but it would quickly converge to a stable state. It was worth mentioning that only the joint motion range and torque limit were considered in the simulation, while the limitation of the hydraulic power unit to the robot’s jumping was not taken into account.

### 5.2. Physical Prototype Experiments

**Figure 13:**
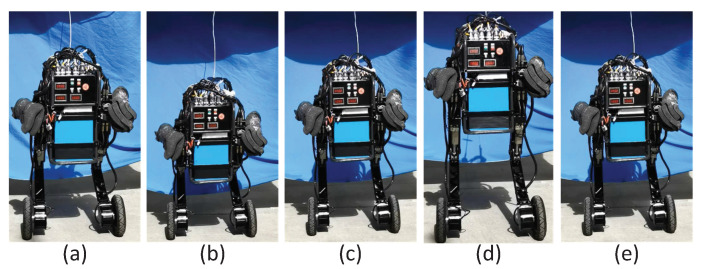
Snapshots of the physical test for changing height. The photos (**a**,**c**,**e**) correspond to the standing height of 0.6 m. Photos (**b**,**d**) correspond to the states with standing heights of 0.5 m and 0.7 m, respectively.

We validated the proposed method on the Scooter prototype mentioned in Section 2. Owing to the limitations of the equipment and robot platform, only the changing height experiment and the velocity tracking experiment were carried out. Figure 13 presents the snapshots of the changing height experiment. The corresponding data curve is plotted in Figure 6. Whether on a simulation platform or a physical prototype, the robot’s standing height could be accurately tracked. However, during the physical test, the pitch angle of the robot torso fluctuated more dramatically. This also caused the wheel torque to vary greatly, and the robot moves back and forth. We believe that the main reason for this phenomenon is that the mass distribution of the torso of the physical prototype is not uniform, and it is difficult to estimate the accurate centroid position. In addition, the bandwidth of the hydraulic actuator is also an important factor, because we achieve the pitch control of the torso through the hip joint.

**Figure 14:**
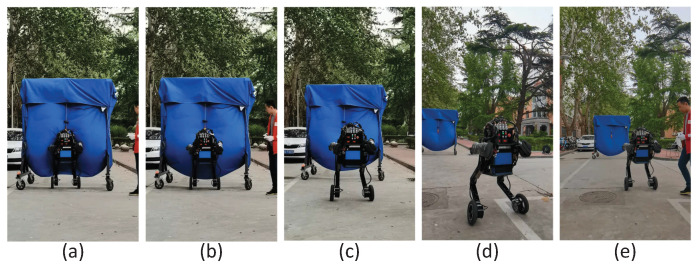
Snapshots of the physical test for velocity tracking. Photos (**a**–**d**) show the forward movement of the robot following a given trajectory. Photos (**d**,**e**) show the process of the robot moving backward due to position overshoot.

Figure 14 presents the snapshots of the velocity tracking experiment. The corresponding data curve is plotted in Figure 10. As could be seen from the data in Figure 10, the physical test results are inferior to those of the simulation. The overshoot in velocity and position was larger and took longer to settle to the desired value. However, the trend of the experimental results was consistent with the simulation results, and the physical prototype also achieved the desired motion. Therefore, we believe that the method proposed in this paper is effective. In addition, the velocity and position curve would appear ahead of the reference curve, because the robot must first ensure the balance of the robot during the movement process.

The experimental results showed that the stable control of the torso was crucial for the WBR, especially when the inertia of the torso was relatively large. This is also an important difference between the WBR and the traditional wheeled inverted pendulum.

## 6. Conclusions and Future Work

In this paper, a decoupling control method of wheeled biped robot was proposed. The dynamic characteristics of the WBR were characterized by two simplified models. The basic balance of the robot was ensured by the TV-LQR controller, and the control of the upper multi-rigid body system is realized by the MPC controller. In the process of constructing the MPC controller, the stability principle of the WBR was fully considered. Through the combination of the above two controllers, the stable movement ability of the WBR was effectively improved. Simulation and experimental results showed that, based on this framework, WBR could realize various motions such as changing height, resisting external disturbances, velocity tracking and jumping. We believe that the method proposed in this paper has certain guiding significance for the control of the WBR, because it simplifies robot modeling and controller building while maintaining control robustness. In the future, the control of the robot in three dimensional cases will be further considered. 

## Figures and Tables

**Table 1:** Parameters of the decoupled model.

Parameter	Description	Value
$m_{w}$	Mass of the wheel	3.5 kg
$I_{w}$	Moment of inertia of the wheel	0.1 kg · m${}^{2}$
*l*	Length of the pendulum	/
θ	Tilt angle of the pendulum	/
φ	Yaw angle of the VL-WIP	/
*r*	Radius of the wheel	0.127 m
*d*	Distance between two wheels	0.63 m
*s*	Displacement of the VL-WIP	/
$\tau_{l}$	Torque about the left wheel	/
$\tau_{r}$	Torque about the right wheel	/
$m_{b}$	Mass of the upper body	73 kg
$I_{y}$	Moment of inertia about the y-axis	$\frac{1}{3}m_{b}l^{2}$
$I_{z}$	Moment of inertia about the z-axis	3.3 kg · m${}^{2}$
$m_{1}$	Mass of the shank	1.2 kg
$m_{2}$	Mass of the thigh	5.3 kg
$m_{3}$	Mass of the torso	60 kg
$l_{1}$	Length of the shank	0.45 m
$l_{2}$	Length of the thigh	0.45 m
$l_{3}$	Height of the torso	0.35 m
$q_{0}$	Angle of the ankle joint	$q_{1}-q_{2}+q_{3}$
$q_{1}$	Angle of the knee joint	/
$q_{2}$	Angle of the hip joint	/
$q_{3}$	Pitch angle of the torso	/

## Supplementary Materials

The following are available online at https://www.mdpi.com/article/10.3390/mi13050747/s1.
